# 12/15-Lipooxygenase Inhibition Reduces Microvessel Constriction and Microthrombi after Subarachnoid Hemorrhage in Mice

**DOI:** 10.21203/rs.3.rs-4468292/v1

**Published:** 2024-06-12

**Authors:** Ari Dienel, Sung Ha Hong, Hussein A Zeineddine, Sithara Thomas, C M Shafeeque, Dania A Jose, Kiara Torres, Jose Guzman, Andrew Dunn, T P Kumar, Gadiparthi N. Rao, Spiros L. Blackburn, Devin W. McBride

**Affiliations:** The Vivian L. Smith, The University of Texas Health Science Center at Houston; The Vivian L. Smith, The University of Texas Health Science Center at Houston; The Vivian L. Smith, The University of Texas Health Science Center at Houston; The Vivian L. Smith, The University of Texas Health Science Center at Houston; The Vivian L. Smith, The University of Texas Health Science Center at Houston; The Vivian L. Smith, The University of Texas Health Science Center at Houston; The Vivian L. Smith, The University of Texas Health Science Center at Houston; The Vivian L. Smith, The University of Texas Health Science Center at Houston; The University of Texas at Austin; The Vivian L. Smith, The University of Texas Health Science Center at Houston; University of Tennessee Health Science Center; The Vivian L. Smith, The University of Texas Health Science Center at Houston; The Vivian L. Smith, The University of Texas Health Science Center at Houston

**Keywords:** subarachnoid hemorrhage, delayed neurological deficit, platelets, 12/15-Lipooxygenase, microvessel constrictions, microthrombi, arterioles

## Abstract

**Background and Purpose:**

Impaired cerebral circulation, induced by blood vessel constrictions and microthrombi, leads to delayed cerebral ischemia after subarachnoid hemorrhage (SAH). 12/15-Lipooxygenase (12/15-LOX) overexpression has been implicated in worsening early brain injury outcomes following SAH. However, it is unknown if 12/15-LOX is important in delayed pathophysiological events after SAH. Since 12/15-LOX produces metabolites that induce inflammation and vasoconstriction, we hypothesized that 12/15-LOX leads to microvessel constriction and microthrombi formation after SAH, and thus 12/15-LOX is an important target to prevent delayed cerebral ischemia.

**Methods:**

SAH was induced in C57BL/6 and 12/15-LOX^−/−^ mice of both sexes by endovascular perforation. Expression of 12/15-LOX was assessed in brain tissue slices and *in vitro*. C57BL/6 mice were administered either ML351 (12/15-LOX inhibitor) or vehicle. Mice were evaluated for daily neuroscore and euthanized on day five to assess cerebral 12/15-LOX expression, vessel constrictions, platelet activation, microthrombi, neurodegeneration, infarction, cortical perfusion, and for development of delayed deficits. Finally, the effect of 12/15-LOX inhibition on platelet activation was assessed in SAH patient samples using a platelet spreading assay.

**Results:**

In SAH mice, 12/15-LOX was upregulated in brain vascular cells and there was an increase in 12-S-HETE. Inhibition of 12/15-LOX improved brain perfusion on days 4–5 and attenuated delayed pathophysiological events, including microvessel constrictions, microthrombi, neuronal degeneration, and infarction. Additionally, 12/15-LOX inhibition reduced platelet activation in human and mouse blood samples.

**Conclusions:**

Cerebrovascular 12/15-LOX overexpression plays a major role in brain dysfunction after SAH by triggering microvessel constrictions and microthrombi formation, which reduces brain perfusion. Inhibiting 12/15-LOX may be a therapeutic target to improve outcomes after SAH.

## Introduction

Subarachnoid hemorrhage (SAH) affects about 30,000 individuals each year in the United States. Up to 30% of patients who survive aneurysm rupture develop delayed cerebral ischemia (DCI) 4 to 10 days following SAH, which accounts for the most common cause of morbidity and mortality in SAH survivors.^1^ Multiple clinical studies indicate the cause of DCI is multifactorial and may include microthrombi, brain blood vessel constriction, and inflammation.^2^

After SAH, there is a significant enhancement in brain arachidonic acid metabolization, which triggers the formation of thrombi and vasospasm, worsening the pathogenesis of SAH.^3,4,5^ 12/15-lipooxygenases (12/15-LOX) are enzymes that catalyze fatty acids, including arachidonic acid and linoleic acid,^6^ into various bioactive lipid metabolites.^7^ Of note, 12/15-hydroxyeicosatetranoic acids (12/15-S-HETE), which are 12/15-LOX metabolites, are potent pro-inflammatory chemoattractants for neutrophils and leukocytes.^8^ 12/15-S-HETE induces the expression of IL-6, IL-12, TNF-α, MCP1, and adhesion molecules in macrophages and vascular cells,^9,10,11^ and disrupts endothelial tight junction and barrier function.^12^ 12/15-LOX has been shown to deleterious in cerebrovascular diseases, such as cerebral ischemia^13,14^ and early brain injury after SAH.^15^ 12/15-LOX is known to trigger inflammation,^16^ activating platelets,^17^ promoting thrombosis^18^ and constricting blood vessels.^19^ As such, 12/15-LOX may cause these events after SAH since it is overactivated after stroke.^20^

Since 12/15-LOX can cause inflammation, thrombi, and vascular dysfunction, and since it is upregulated after SAH, we hypothesized that inhibiting 12/15-LOX after SAH will reduce microvessel constrictions and formation of microthrombi, thus improving brain perfusion and preventing DCI after SAH in mice.

## Materials and Methods

Study approval: All procedures performed on animals were approved by the UTHealth Animal Welfare Committee and conducted according to the NIH Guide for the Care and Use of Laboratory Animals. The results are reported in accordance with ARRIVE (Animal Research: Reporting in Vivo Experiments) guidelines.

Animal Study: Two hundred thirty adult C57BL/6 and fifty-five 12/15-LOX^−/−^ mice (4–6 months old) of both sexes were used. Mice were housed under a 12-h day/12-h night cycle with free access to food and water. SigmaPlot 11.0 was used to estimate all sample sizes using data from previous experiments and preliminary data with α = 0.05 and β = 0.2.^21^ C57BL6 mice were electronically randomized into either Sham, SAH, Sham + Vehicle, SAH + Vehicle, or SAH + ML351, while 12/15-LOX^−/−^ mice were randomized into sham or SAH. The same individual performed all surgeries and investigators responsible for functional assessment, outcome measurement, and data analysis were blinded to experimental groups, sex, and genotype.

Cerebral blood flow (CBF) and intracranial pressure (ICP) were monitored as previously described^22,23,24^ using a laser Doppler probe (Perimed, Järfälla, Sweden) and microcatheter transducer (Millar, Houston, USA), respectively. SAH was induced using the endovascular perforation model as described previously.^21^ In brief, a 5 – 0 monofilament filament was inserted into the left external carotid artery and advanced towards the Circle of Willis. After confirming SAH induction (an immediate CBF drop of at least 85% or an immediate ICP increase from 1–3 mmHg to 40–80 mmHg), the filament was immediately removed, and the external carotid artery was ligated. Sham-operated animals were treated similarly, with the exception that the filament was not advanced far enough to induce SAH. After recovery from isoflurane, mice were observed for up to four hours and only mice without hemiparesis were included in this study. Mice were allowed to survive for up to 5 days post-SAH. Health status was assessed a minimum of three times per day. Mice not surviving from day 1 to day 5 were excluded from all outcomes except mortality, behavior, laser speckle imaging, and delayed neurological deficits (DND). Excluded mice were replaced to satisfy sample size calculations for the primary outcomes (microvessel constriction, microthrombi, infarction, and 12-S-HETE levels).

Fifteen minutes after SAH induction (and in selected sham mice), C57BL/6 mice were given an intravenous injection of either vehicle or 25mg/kg ML351 (12/15-LOX antagonist, 531492, Sigma-Aldrich). The vehicle consisted of saline containing 10% Chremophor EL (5135, Sigma-Aldrich), 10% Solutol HS 15 (42966–1KG, Sigma-Aldrich), and 20% PGE400 (25322-68-3, Sigma-Aldrich).

Western blot: Sham or SAH C57BL/6 mice (n = 6/group/time-point) were euthanized via PBS perfusion on days 1 or 5. Brains were homogenized and 60 μg of protein was loaded into the wells. Nitrocellulose membranes were incubated with 12-LOX (1:250, sc-365194, Santa Cruz), and β-Actin (1:1000, sc-47778, Santa Cruz) overnight at 4°C, followed by incubation with HRP-conjugated secondary antibodies (1:1000, sc-516102, Santa Cruz) for 1 hour at room temperature. Nitrocellulose membranes were imaged (Imager, Azure Biosystems). Using ImageJ, protein bands were normalized to β-actin for each lane and then normalized to sham values for each membrane.

### ELISA

Sham or SAH C57BL/6 mice (n = 5/group/sex/time-point) were euthanized at day 1, 2, 5, or 6 post-SAH for assessment of 12-S-HETE plasma levels. A separate cohort of mice (n = 5/group/sex for C57BL/6 mice, n = 5/group for male 12/15-LOX^−/−^ mice) was euthanized on day 5 to assess the effect of 12/−15-LOX inhibition/knockout on 12-S-HETE plasma levels. Blood was collected via cardiac puncture into a syringe containing 50μL 3.8% citric acid. Blood was centrifuged at 2000g for 15 minutes to obtain plasma. Plasma was stored at −80°C until measurement. 12-S-HETE concentration was measured using an ELISA kit (ab133034, Abcam) according to manufacturer guidelines.

Immunostaining: One sham and one SAH male C57BL/6 mouse were euthanized on day 5 via cardiac perfusion of PBS and the brains were stored in 3% glyoxal until processing.^25^ Brains were sectioned into 40μm thick slices using a vibratome (Leica VT 1000S). Brain slices (at −2 from bregma) were stained for 12-LOX (1:100, sc-365194, Santa Cruz), 15-LOX (1:100, sc-133085, Santa Cruz), and laminin (1:200, sc-59854, Santa Cruz). Briefly, through free-floating technique, brain slices were permeated in 0.3% Triton for 30 min and blocked with 3% BSA. Thereafter, brain slices were incubated with the primary antibodies overnight at 4°C. Incubation with the second antibodies followed and lasted 1hour at room temperature.

The samples were carefully placed onto microscope slides using Fluoromount-G (0100–01, SouthernBiotech). Slices were observed for 12-LOX, 15-LOX, and laminin expression using a microscope and images were processed using THUNDER (Leica DMI8 Thunder microscope).

Microvessel constrictions: Five days after SAH, mice (n = 6–8/group/sex/strain) were euthanized via cardiac perfusion of PBS followed by gelatin-India ink (1:2 India ink:10% gelatin). Mice were stored at 4°C overnight, then brains were removed and stored in 4% PFA for 48 hours then stored in PBS with 0.01% sodium azide until imaging. A Zeiss Discovery stereomicroscope was used to image the entire surface of the brain. Vessel constriction was evaluated by determining locations in the artery segments with had a diameter reduction of more than 10%. Large arteries and arterioles were defined as > 50μm and 10–50μm, respectively.

### Platelet morphology

Prior to processing blood samples, coverslips (12-545-101P, Fisher Scientific) were coated with poly-L-lysin (0.1% (v/v) in PBS, 0413, ScienCell) solution and incubated for 30 min at 37°C in 35mm tissue culture dishes (130180, Thermo Scientific). Thereafter, cover glasses were washed with PBS and dried in air for 30 min. Slides were stored in a dust-free box until use.

Blood was collected from mice (n = 6/group/sex/strain) on day 5 via cardiac puncture into a syringe containing 50μL ACD buffer (8013-89-6, Sigma Aldrich). The blood was mixed with wash buffer (2:1 ratio, buffer contained 10 mM sodium citrate, 150 mM NaCl, 1 mM EDTA, and 1% (w/v) dextrose in Tyrode’s buffer (11760–10, EMS) at pH 7.4) and centrifuged for 5 min at 800g and room temperature. The supernatant (platelet-rich plasma) was collected and then centrifuged for 20 min at 100g and room temperature. The supernatant was collected and centrifuged again for 20 min at 800g and room temperature. The supernatant was carefully aspirated until about 100 μL of the solution remained with the pellet. Then 1400 μl of glucose solution (0.1% glucose + 0.3% BSA dissolved in Tyrode’s buffer) was added to the sample.

The tubes were flicked three times and then incubated at 37°C for 15 min. Each sample was placed onto the prepared poly-L-lysin coated glass slides, followed by incubation for 30 min at 37°C. Slides were then washed 2 times with PBS. After aspiration of PBS, the platelet samples were fixed with 4% PFA for 15 min. PFA was aspirated, samples were washed, then 0.3% Triton-X100 was added for 10 min, following by incubation with Phalloidin-647 (1:1000, ab176759, Abcam) for 2 hours. In select samples a CD42a antibody (1:1000, sc-166420, Santa Cruz Biotechnology) was also added to confirm the cells were platelets. Samples were washed and then allowed to dry in the dark overnight at room temperature. The samples were carefully placed onto microscope slides using Fluoromount-G.

Images were taken over the whole area using a fluorescence microscope at 100x. Platelet morphology was determined by a blinded investigator for each image. Platelets were categorized as either inactiveted (discoidal shaped) or activated (spikey, partially spread, or spread) based on morphology.^26,27^

### Microthrombi

On day 5 post-SAH, mice (n = 6/group/sex/strain) were administered heparin (50μL/10g, 1000U/mL) 5 min before euthanasia which consisted of cardiac perfusion of PBS followed by 4% PFA. Brains were removed and stored in PFA in a 4°C refrigerator before sectioning into 40μm thick slices. Slices at −2 from bregma were stained with Martius Scarlet and Blue (MSB) and microthrombi were counted throughout the entire slice as previously described.^21^

### Neurodegeneration

Fixed brain slices (n = 6/group/sex/strain) at −2 bregma were stained with Fluorojade C following the manufacturer’s protocol (AG325, Millipore). Neurons undergoing degeneration (*i.e*. exhibited positive staining) were counted throughout the entire slice using a Leica DMi8 microscope.

### Infarction

Mice (n = 6–8/group/sex/strain) euthanized on day 5 had one fixed brain slice between − 1 and – 2 from bregma stained with crysel violet at room temperature following manufacturer methods. The entire slice was imaged to quantify the total area of infarcted tissue.

### Laser speckle contrast imaging of brain perfusion

Male mice (n = 6/group/strain) were used to study cortical brain perfusion using Laser Speckle Contrast imaging. Mice received a cranial window implantation of stacked coverglass (three 3mm glasses stacked on a 5mm glass) 3–4 weeks before SAH. Briefly, the skull was exposed in anesthetized mice. Then a 4mm diameter circle was created using a microdrill, ensuring that the edges were at least 1mm away from the midline and the bregma/lambda sutures. After skull removal and bleeding cessation, the stacked glass coverslip was placed into the hole after being filled with aCSF. Dental cement was applied around the stacked 5mm glass edge to secure it to the skull. A custom-made head frame was attached using super glue and dental cement.^28^ The mice were allowed to recover at least 3 weeks before SAH.

On the day of SAH, the cranial windows were examined and mice which had windows that were opaque or not secure were excluded and replaced. Brain perfusion data was collected for 10 min before SAH (baseline perfusion) and for up to 80 min post-SAH using an in-house built laser speckle imaging system.^29^ At 15 min after SAH, recording was paused to inject ML351 or vehicle. Imaging was also performed for 10 min on days 1–5. Data was analyzed using the MatLab algorithms developed by the Functional Optical Imaging Laboratory.^29,30^ In brief, ROIs were drawn to measure cerebral perfusion of the MCA, ACA, and watershed territories. Within each ROI, the cerebral perfusion units were normalized to the baseline perfusion units and are presented as percent of baseline.

### Neurobehavior and DND

All mice surviving more than 1 day are included in neurological assessment. Daily behavioral performance was assessed 1–5 days post-SAH using an 8-test sensorimotor neuroscore which evaluates functional performance in exploration, climbing, forelimb and hind limb use, whisker and side sensation, balance, and visual reflex.^31^ In brief, the maximum score of 24 corresponds to no deficits and the minimum score of 0 is unresponsive.

DND is classified as mice experiencing a reduction of more than 4 points in the neuroscore after recovery from the day 1 neuroscore. Delayed death on days 3–5 (if some functional recovery was observed on prior days) is also considered as developing DND as the neuroscore would be 0. Mice which exhibited continuous neuroscore decline from day 1 are not included in DND analysis.

in-vitro study: To determine which vascular cells expressed 12/15-LOX, we cultured brain microvascular endothelial cells from human (HBMVEC, ACBRI-376, Cell Systems) and mouse (MBMVEC, C57–6023, Cell Biologics), and human brain vascular pericytes (ACBRI-498, Cell Systems). Cells were grown in DMEM-F12 supplemented with 10% FBS (A5256801, Gibco), 1% antibiotic-antimycotic (CA002–010, GenDepot), and 0.2% Normocin (NC9273499, Fisher Scientific). Upon reaching 70% confluency, cells were collected and sonicated. Protein expression was quantified using Western blot. Briefly, membranes were incubated with 12-LOX (1:250, sc-365194, Santa Cruz), 15-LOX primary antibodies (1:100, sc-133085, Santa Cruz), or β-Actin (1:1000, sc-47778, Santa Cruz) overnight at 4°C, incubated with HRP-conjugated secondary antibody (1:1000, sc-516102, Santa Cruz) for 1 hour at room temperature, and imaged as described in the Western blot subsection. Membranes were striped for each of the three primary antibodies.

Human Study: All procedures performed on human samples were approved by UTHealth and conducted according to the NIH Guidelines. Aneurysmal SAH patients and control human blood were enrolled (via written informed consent prior to participation) into the study under a protocol approved by the UTHealth Institutional Review Board. Blood was collected from fourteen aneurysmal SAH patients (confirmed via CT angiography) on days 1, 2, 4, and 7 post-rupture, and from nine control humans, into BD Vacutainer ACD-B (364816, Becton Dickinson and Company) between 9:00a and 4:00p. Blood was processed within 30 min after it was drawn. Briefly, the blood was mixed with wash buffer (1:1 ratio) in a 15mL falcon tube and processed as described in the “platelet morphology” outcome. After the three centrifugation steps as described in “platelet morphology”, supernatant was vacuum sucked and platelet washing buffer (1500μL) was added to the pellet, and the tube was flicked three times to resuspend the pellet. The solution was then centrifuged for 8 min at 100g and room temperature. Thereafter, the supernatant was collected and platelets counted (Hemavet 950 FS, Drew Scientific). Following platelet counting, 750μL of platelet sample was put into two tubes. One tube had 100μL of ML351 (final concentration of 10μM) added and the other tube had 100μL of vehicle (10% solutol, 10% Chremophor EL, and 20% PGE400 in 0.9% NaCl saline) added. The tubes were flicked three times and then incubated at 37°C for 15 min. Then each sample was placed onto the prepared poly-L-lysin coated glass slides, and processed as described in the “platelet morphology” outcome.

### Statistical analysis:

Unless otherwise specified, data are presented as mean and SD with individual values. All outcomes were tested for normality and homoscedasticity, and if failed, the equivalent non-parametric tests were used. Multiple groups were analyzed using one-way ANOVA with Tukey post hoc or Kruskal-Wallis with Dunn’s post hoc. Laser speckle contrast imaging data was analyzed using two-way ANOVA. Neuroscore data was analyzed using two-way ANOVA on ranks (Friedman) followed by Wilcoxon signed-rank posthoc then corrected using a Bonferroni correction. DND incidence was analyzed using a log-rank test. Unpaired t-tests were used to compare sex differences, differences between 12/15-LOX^−/−^ Sham and SAH, and for the human platelet morphology outcome. All calculations were performed using a SPSS v28 and Graphpad Prism 6. Differences were considered to be significant at p< 0.05.

## Results

For C57BL/6 the mortality rate from days 1 to 5 post-SAH was 0/42 for both male and female sham and sham + vehicle groups, 18/71 male and 9/42 female SAH (+ vehicle) mice, and 3/37 male 8/34 female SAH + ML351 mice. For 12/15-LOX^−/−^ mice the mortality rate from days 1 to 5 post-SAH was 0/25 for both male and female sham groups and 3/16 male SAH and 3/14 female SAH.

### SAH causes increased 12/15-LOX expression in brain vasculature

**Figure 1:**
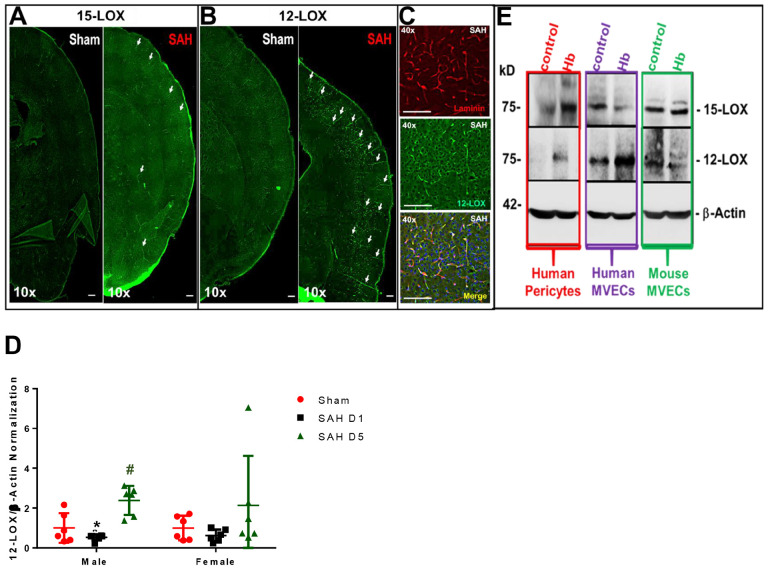
Expression of 12/15-LOX. (**A-C**) Immunostaining for 12- and 15-LOX in brains from male mice 5 days after SAH. 12/15-LOX is green, laminin (vasculature) is red. Scale Bar = 50 μm. **(D)** Quantification of 12-LOX protein. n=6/group/sex. One-way ANOVA with Tukey. *p<0.05 vs. Sham, ^#^p<0.05 vs. SAH D1. (**E**) Protein expression (Western blot) of 12- and 15-LOX and β-Actin in human brain pericytes, human brain microvascular endothelial cells, and mouse brain microvascular endothelial cells.

Before examining the impact of 12/15-LOX on SAH outcomes, we stained the brain for 12/15-LOX to identify where 12/15-LOX is expressed and if there are changes after SAH. Using immunostaining of male brains, we observed that SAH induces an increase in 12/15-LOX expression in the brain vasculature on day 5 post-SAH (Fig. 1A–C). The cortical vessels had the highest expression of 12/15-LOX. In male mice, the 12/15-LOX expression was significantly more elevated on day 5 compared to day 1 (Sham vs SAH D5 p = 0.0034 and SAH D1 vs SAH D5 p = 0.0003, Fig. 1D). Female mice did not have a statistically significant increase in brain 12/15-LOX expression in female mice (Fig. 1D). As 12/15-LOX expression was upregulated in the brain vasculature, to identify if brain endothelial cells or pericytes have more expression after SAH, we performed cell culture of human and mouse brain microvascular endothelial cells and human pericytes subjected to hemoglobin toxicity. Western blot analysis of 12/15-LOX expression by pericytes and microvascular endothelial cells indicates increased expression of 12/15-LOX after hemoglobin injury (Fig. 1E).

#### 12-S-HETE is elevated on day 5 and is attenuated by 12/15-LOX inhibition

**Figure 2:**
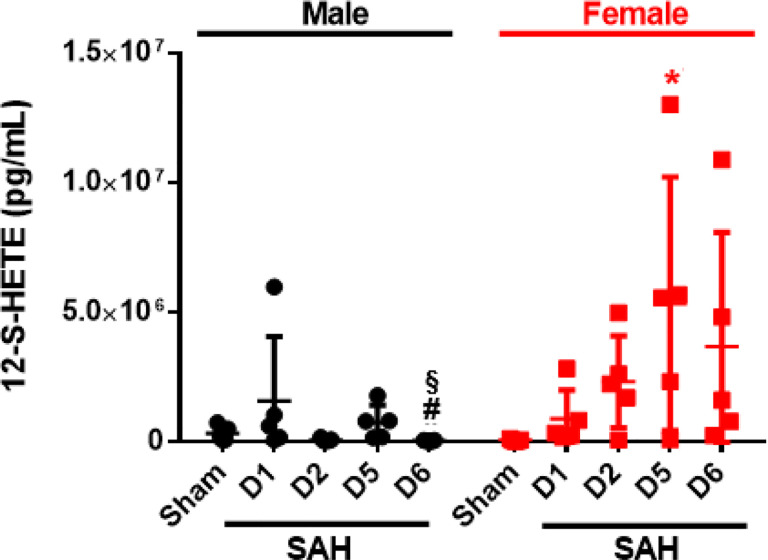
Plasma 12-S-HETE levels after SAH. n=5/group/sex. Kruskal-Wallis with Dunn’s test. *p<0.05 vs Sham, ^#^p<0.05 vs SAH D1, ^§^p<0.05 vs SAH D5.

12/15-S-HETE is a primary metabolite generated by 12/15-LOX, and since 12/15-S-HETE has known inflammatory and pro-constrictive properties, we measured the plasma levels of 12-S-HETE following SAH in mice. In female mice, 12-S-HETE increases gradually peaking on D5 (p = 0.0128 vs Sham), while 12-S-HETE levels in male mice show a tendency towards higher levels (p = 0.1127, for D5 vs D2). Interestingly, SAH causes females to have significantly higher 12-S-HETE levels than males (D2: p = 0.0223, D5: p = 0.0716) (Fig. 2).

**Figure 3:**
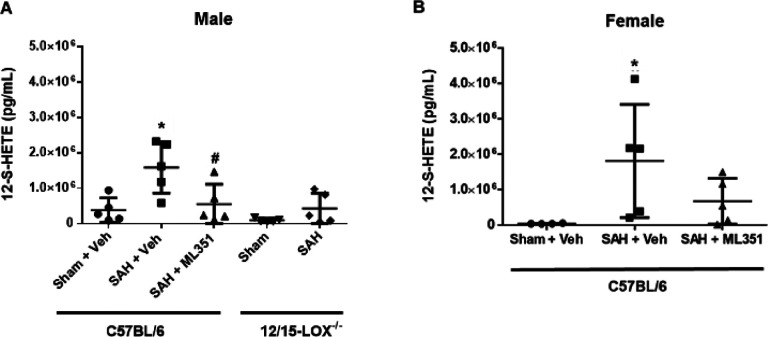
12/15-LOX inhibition reduces plasma 12-S-HETE on post-SAH Day 5. **(A)** Male mice. **(B)** Female mice. n=5/group/strain/sex, except for female Sham + Veh which n=4. One-way ANOVA test with Tukey (male) and Dunn’s multiple comparison test (female), unpaired t-test for 12/15-LOX^−/−^ mice. *p<0.05 vs Sham + Veh or Sham, ^#^p<0.05 vs SAH + Veh. One data point for the Sham + Veh group was excluded since it was more than 7800% from the mean.

As 12-S-HETE levels are elevated after SAH, we then examined if inhibition of 12/15-LOX by ML351 could attenuate the 12-S-HETE plasma levels on day 5. In males, 12-S-HETE concentration is significantly higher in SAH + Vehicle when compared to Sham + Vehicle (p = 0.0144), which was significantly reduced by ML351 treatment (p = 0.0338, Fig. 3A). Five days after SAH, 12/15-LOX knockout mice also had significantly lower 12-S-HETE levels than C57BL/6 mice (p = 0.0158). In female mice, 12-S-HETE is increased in SAH + Vehicle (p = 0.0279 vs Sham + Vehicle), with no effect towards reduction of 12-S-HETE after treatment with ML351 (p = 0.5965) (Fig. 3B).

### 12/15-LOX inhibition reduces microvessel constrictions on day 5

**Figure 4:**
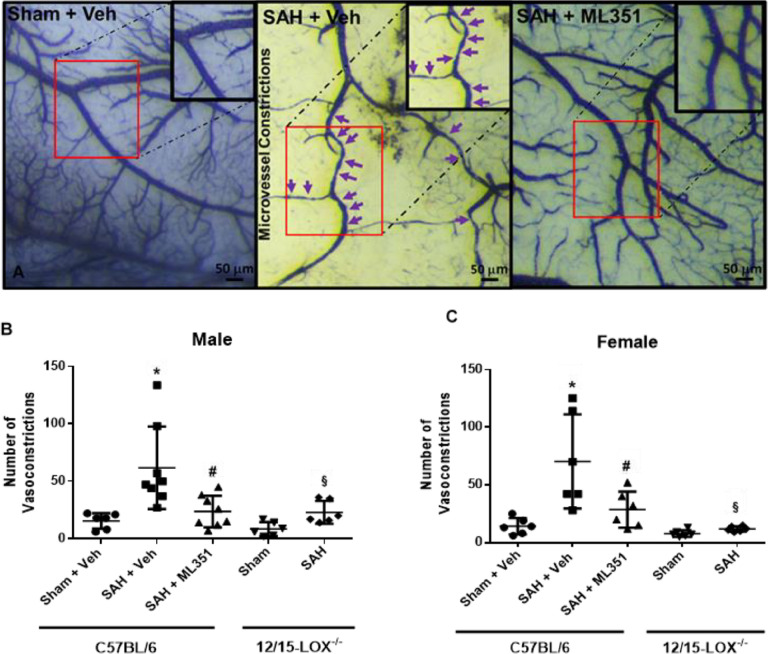
12/15-LOX inhibition reduces microvessel constrictions on post-SAH Day 5. **(A)** Representative images of microvessel constrictions in the brain. **(B, C)** Quantification of constrictions in arterioles. n=6–8/group/sex. One-way ANOVA with Tukey post-hoc. *p<0.05 vs Sham + Veh, ^#^p<0.05 vs SAH + Veh. Unpaired t-test for 12/15-LOX^−/−^ mice: ^§^p<0.05 vs 12/15-LOX^−/−^-Sham.

Since 12-S-HETE is a vasoconstrictor,^19^ we investigated if ML351 could attenuate brain blood vessel constriction after SAH. Brain vessels were visualized using gelatin-India ink and the number of vessels exhibiting constriction were counted. On day 5, SAH leads to a significant number of microvessel constrictions (10–50μm) which is attenuated by inhibition of 12/15-LOX in male and female mice (male: p = 0.0121, female: p = 0.0321). Similarly, significantly fewer microvessel constrictions were observed in 12/15-LOX knockout SAH mice compared to C57BL/6 mice with SAH (male: p = 0.0185, female: p = 0.0008) (Fig. 4).

### 12/15-LOX inhibition reduces platelet activation and microthrombi formation on day 5

**Figure 5:**
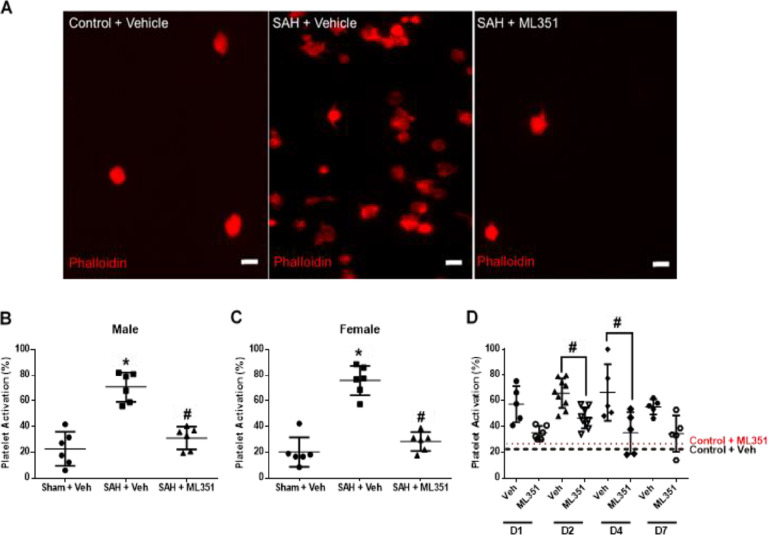
12/15-LOX inhibition reduces platelet activation after SAH in mice and humans. **(A)** Representative images of mouse platelet morphology. Scale bar = 5μm. **(B-C)** Quantification of platelet activation for mice. One-way ANOVA with Tukey post-hoc. n=6/group. *p<0.05 vs Sham + Veh and ^#^p<0.05 vs SAH + Veh. **(D)** Quantification of platelet activation for SAH patients. Lines show the mean values of platelet activation from control patient’s blood treated with vehicle (black dashed) or ML351 (red dotted). Unpaired t-test. n=5–9/group/time-point. ^#^p<0.05.

As 12-S-HETE is also known to promote activation and aggregation of platelets,^17,18^ and since microthrombi are reported to be part of DCI after SAH,^21^ we measured the effect of 12/15-LOX inhibition on platelet morphology changes and brain microthrombi. As assessed using the platelet spreading assay, 5 days after SAH, platelets are significantly activated in SAH + Vehicle mice compared to Sham + Vehicle mice (male: p < 0.0001; female: p < 0.0001) (Fig. 5A–C). SAH mice treated with ML351 showed a significant reduction in activated platelets compared to SAH mice treated with vehicle (male: p < 0.0001; female: p < 0.0001).

To test human relevance of platelet activation after SAH, and to test if 12/5-LOX inhibition could alter platelet morphology changes, we assessed platelet spreading in human platelets at several time-points post-SAH. Compared to control patient blood, SAH induces a significant increase in platelet activation for up to 7 days post-SAH (Fig. 5D). Since platelets use 12-S-HETE to self-regulate *(i.e*. promote activation via 12-S-HETE release),^32^ we examined if ML351 could prevent platelet spreading. Human SAH platelets treated with ML351 showed significantly less platelet activation than platelets treated with vehicle 2 and 4 days after SAH (D1: p = 0.091, D2: p = 0.0399, D4: p = 0.0062, D7: p = 0.1749) (Fig. 5).

**Figure 6:**
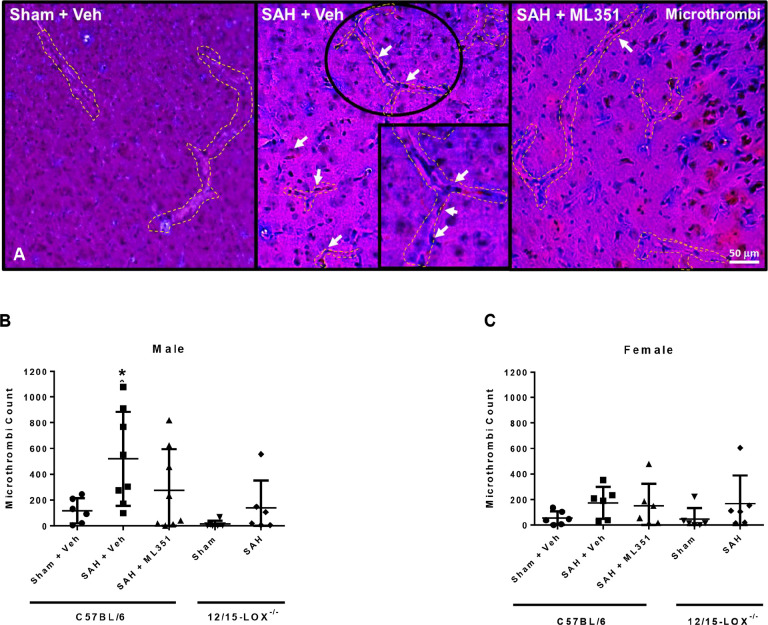
12/15-LOX inhibition reduces microthrombi on Day 5 after SAH. **(A)** Representative image of microthrombi in the brain of a SAH mouse. Scale bar: 50μm. **(B, C)** Quantification of microthrombi in mice. n=6/group/sex/strain. One-way ANOVA with Tukey post-hoc. *p<0.05 vs Sham + Veh. Unpaired t-Test for 12/15-LOX^−/−^ mice.

While platelet spreading is reduced by 12/15-LOX inhibition, we also investigated if inhibiting 12/15-LOX could attenuate microthrombosis after SAH. In MSB stained of brain slices from mice euthanized on day 5, microthrombi were visible in the cerebral vasculature of SAH male mice (male: p = 0.0202 vs Sham + Vehicle), but not females (p = 0.2688 vs. Sham + Vehicle). Treatment with ML351 partially reduced the microthrombi counts in male SAH mice compared to vehicle treatment (p = 0.2715). However, 12/15-LOX^−/−^ males with SAH had significantly less microthrombi than C57BL/6 male SAH + Vehicle mice (p = 0.0147) (Fig. 6).

### 12/15-LOX inhibition reduces neurodegeneration on day 5

**Figure 7:**
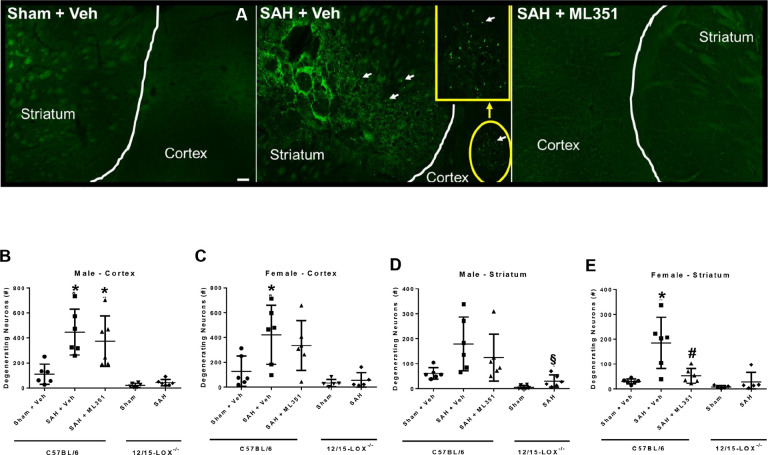
12/15-LOX inhibition with ML351 reduces neurodegeneration on Day 5 after SAH. **(A)** Representative image of neurodegeneration. **(B-E)** Quantification of neuronal degeneration in the brain cortex **(B, C)** and striatum **(D, E)**. n=6/group/sex/strain. One-way ANOVA with Tukey post-hoc. *p<0.05 vs Sham + Veh, ^#^p<0.05 vs SAH + Veh. Unpaired t-test for 12/15-LOX^−/−^ mice: ^§^p<0.05 vs 12/15-LOX^−/−^-Sham.

We next measured neuronal neurodegeneration with Fluorojade-C. SAH caused significant neuronal death (SAH + Vehicle vs Sham + Vehicle: male p = 0.0077, female p = 0.0460) ML351 significantly attenuated neuronal degeneration in the striatum of female mice (p = 0.0057), but not male mice (p = 0.5068). However, 12/15-LOX^−/−^ mice with SAH have significantly less neuronal degeneration than wild-type mice with SAH (male: p = 0.0001; female: p = 0.0058). In the striatum, 12/15-LOX^−/−^ mice with SAH had significantly less neuronal degeneration than wild-type mice with SAH (male: p = 0.0078; female: p = 0.0109) (Fig. 7).

### 12/15-LOX inhibition improves brain perfusion on days 4 and 5

**Figure 8:**
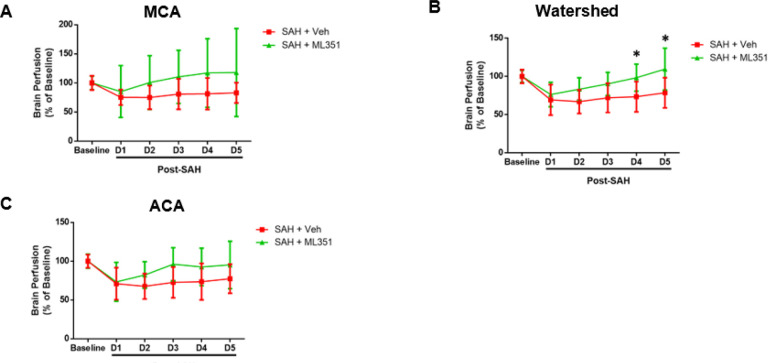
Inhibition of 12/15-LOX improves brain perfusion in the watershed area. n=6/group/sex/strain. Two-way ANOVA with Sidak’s multiple comparison test. * p<0.05 vs SAH + Veh.

Following SAH, brain perfusion becomes compromised. To examine if 12/15-LOX inhibition could restore the lessened brain perfusion, we performed laser speckle contrast imaging in male mice and evaluated the MCA, ACA, and watershed territories via a cranial window. While the MCA and ACA areas had little improvement in perfusion with ML351 treatment, the watershed region displayed significantly improved brain perfusion in treated mice on days 4 (p = 0.0457 SAH + Vehicle vs SAH + ML351) and 5 (p = 0.0063 SAH + Vehicle vs SAH + ML351) (Fig. 8).

### 12/15-LOX inhibition improves neurological behavior and prevents the development of DND

**Figure 9:**
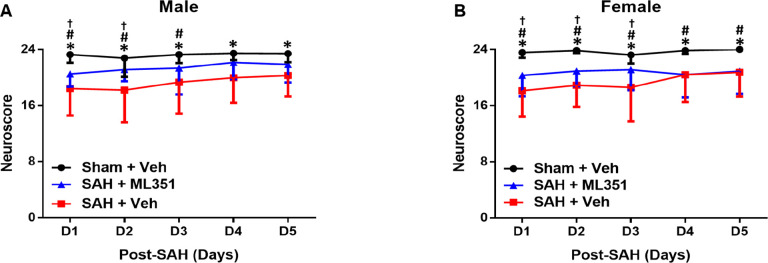
12/15-LOX inhibition ameliorates neurological deficits in SAH mice. n=25–41 male/group/time-point and n=25–30 female/group/time-point. Friedman ANOVA followed by Wilcoxon signed-rank posthoc then Bonferroni correction. *p<0.05 vs Sham + Veh vs SAH + Veh, ^#^p<0.05 vs Sham + Veh vs SAH + ML351, ^†^p<0.05 vs SAH + Veh vs SAH + ML351.

Neurological behavior was assessed using a neuroscore each day. Vehicle-treated mice with SAH had significant neuroscore deficits compared to Sham + Vehicle, whereas ML351 treated mice had improved behavior at day 1 (male p = 0.024, female p = 0.105) which remained for at least 3 days (Fig. 9).

**Figure 10:**
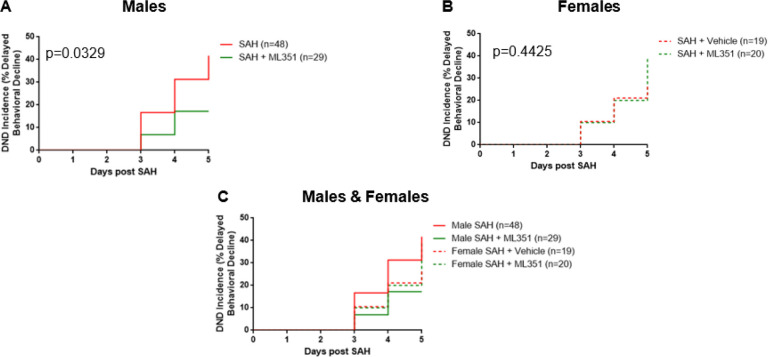
DND incidence. Data from all mice of the same sex were combined for DND analysis. SAH and SAH + Vehicle male mice were combined to increase power since there was no difference in DND incidence between these two injury control groups (Supplemental Fig 6). Log-rank (Mantel-Cox) Test.

**Figure 11:**
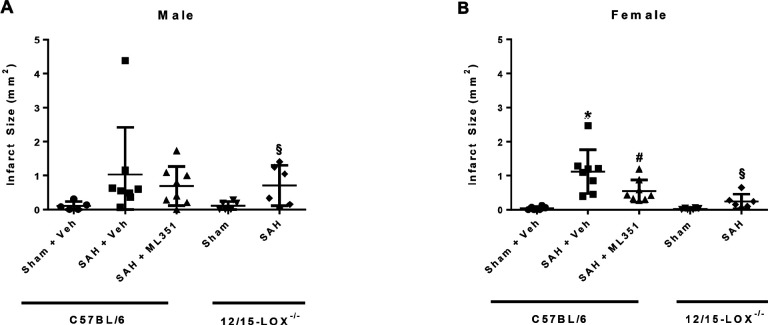
Day 5 infarct area after SAH. n=6–8/group/sex/strain. One-way ANOVA with Tukey post-hoc. *p<0.05 vs Sham + Veh, ^#^p<0.05 vs SAH + Veh. Unpaired t-test for 12/15-LOX^−/−^ mice: ^§^p<0.05 vs 12/15-LOX^−/−^-Sham.

As DCI is a major contributor to poor outcome for SAH, we measured cerebral infarction on day 5 and assessed for the development of delayed neurological deficits which are hallmarks of clinical DCI (Fig. 10). The infarct volume is significantly higher in SAH female mice than sham (p = 0.0032) which can be significantly reduced by ML351 treatment (p = 0.0457). However, there was no significant infarction in male SAH mice (Sham + Vehicle vs SAH + Vehicle p = 0.1622) and no significant reduction of infarction after treatment with ML351 (p = 0.6405 vs SAH + Veh) (Fig. 11). Inhibition of 12/15-LOX by ML351 lead to a significant reduction in DND incidence for male SAH mice (p = 0.0329) but not for female SAH mice (p = 0.4425). There is also a tendency for less DND incidence in male ML351-treated mice as compared to female ML351-treated mice (p = 0.0714) (Fig. 10).

## Discussion

Approximately 30% of patients who survive aneurysm rupture develop DCI 4 to 10 days post-SAH, which is the major cause of morbidity and mortality among SAH survivors.^1^ Various clinical studies indicate that the cause of DCI is multifaceted, involving factors such as microthrombi, vasospasm, and inflammation.^2^ In this study, we examined the role of 12/15-LOX in delayed microvessel constrictions and microthrombi after SAH. For the first time, we observed that 1) 12/15-LOX is upregulated in brain microvessels after SAH, 2) a 12/15-LOX metabolite, 12-S-HETE, expression peaks day 5, 3) inhibiting 12/15-LOX can reduce delayed vasospasm, microthrombi, and neuronal degeneration, resulting in improved cerebral perfusion which ultimately improved outcomes following SAH (including reducing DND incidence), 4) platelet spreading is increased by SAH and inhibition of ML351 can prevent platelet spreading in humans and mice, and 5) there are sex differences in the response to ML351 treatment. These findings collectively demonstrate a critical role of 12/15-LOX in the pathophysiology of SAH and warrants investigation to determine its potential as a therapeutic target to mitigate the adverse effects of SAH.

Following an insult, such as SAH, arachidonic acid is released from cell membranes by phospholipases^33^ and arachidonic acid metabolism leads to metabolites which promote inflammation.^34^ Arachidonic acid metabolism is enhanced, especially within the brain cortex, after SAH via both the cyclooxygenease and lipooxygenase pathways which may be responsible for arterial constrictions and blood clot formation.^3^

It was recently reported that 12/15-LOX is involved in early brain injury after SAH. Specifically, 12/15-LOX was overexpressed in macrophages triggering inflammation leading to edema formation and neuronal cell death.^15^ Other studies have suggested that early brain injury may be a factor involved in causing DCI,^35,36^ so we sought to investigate if 12/15-LOX could be a link between early brain injury and DCI. Herein, we observed that whole brain levels of 12/15-LOX are relatively unchanged at 1 day, but significantly elevated on day 5 (Fig. 1E, Supplemental Fig. 1). We also observed a high expression of 12/15-LOX within the penetrating arterioles of the brain cortex on day 5, which is likely due to increased expression by microvascular endothelial cells (Fig. 1A–C, Supplemental Fig. 1A-E).

12/15-S-HETE, which induces the expression of many inflammatory markers and adhesion molecules in macrophages and vascular cells,^9,10,11^ and disrupts endothelial tight junction and barrier function,^37^ is highly increased in blood plasma during the DCI phase. This increase may be a cause of delayed vessel constriction and microthrombi formation, potentially to its impact on platelets.

While large vessel vasospasm can be prevented with various pharmacological interventions after SAH,^38,39^ DCI incidence remains high. There are limited studies identifying potential mechanisms responsible for microvessel constrictions, which are known to be present after SAH.^40^ As 12/15-LOX is elevated in microvessels, and as no study has addressed the impact of 12/15-LOX on the formation of cerebral small vessel constriction and microthrombi after SAH nor 12/15-LOX involvement in brain perfusion, we explored the pathological interaction between 12/15-LOX, microvessels, and platelets. We found that increased 12/15-LOX metabolites, as 12-S-HETE, may play an important role in platelet activation and small vessel constrictions, impacting brain perfusion, since 12/15-LOX inhibition attenuated these pathophysiological events. Despite providing reduced microvessel constriction and platelet spreading in females, only male mice exhibited a reduction in the DND incidence. There are several potential reasons. First, the higher 12-S-HETE levels in females even after ML351 treatment may impede recovery from SAH. Second, we chose a treatment regimen that worked to prevent EBI in male mice with SAH. So, it may be necessary to use a different treatment regimen (dosing, timing) better suited for female mice to address this disparity.

In patients developing DCI, cerebral perfusion is thought to be reduced in localized areas, leading to infarction or DND. To examine if 12/15-LOX impacts brain perfusion, we performed laser speckle contrast imaging. We found that inhibiting 12/15-LOX can significantly improve the perfusion of the watershed areas but had no effect on the large artery perfusion. Since watershed areas make up around 10% of all brain infractions after ischemic stroke,^41^ they may also play an important role for SAH. The cause of improved watershed area perfusion by 12/15-LOX inhibitions remains unknown, but may be due to fewer microthrombi or less microvessel constrictions. Regardless, if 12/15-LOX inhibition improves watershed perfusion after SAH, it may be a therapeutic target to improve watershed perfusion for cerebrovascular diseases.

### Clinical Implications

It is well-known that arachidonic acid metabolism is deleterious following cerebrovascular diseases, including SAH.^42^ Arachidonic acid can be metabolized by three major classes of enzymes, namely cytochrome P450, cyclooxygenase (COX), and LOX.^43^ COX, which promotes inflammatory response and platelet function,^44,45,46,47,48^ may not be able to decrease the incidence of DCI. COX inhibitors, such as aspirin, have been tested in clinical studies and overall do not decrease the incidence of DCI.^49^ Cytochrome P450 is also unlikely to be a major factor in arachidonic acid metabolism following SAH as there are low brain levels of cytochrome P450 and restricted expression.^50,51^ Together with the study by Gaberel *et al*.,^15^ 12/15-LOX may be a key player in the metabolism of arachidonic acid after brain injury. But one caveat to note is that arachidonic acid can be metabolized by any one of these three enzymes. Thus, inhibiting one enzyme may increase the metabolism of arachidonic acid by another enzyme, thereby producing more of its metabolites. So a question that remains is can inhibition of a single arachidonic acid enzyme be enough to promote recovery or do COX and LOX need to be inhibited simultaneously?

**Figure 12:**
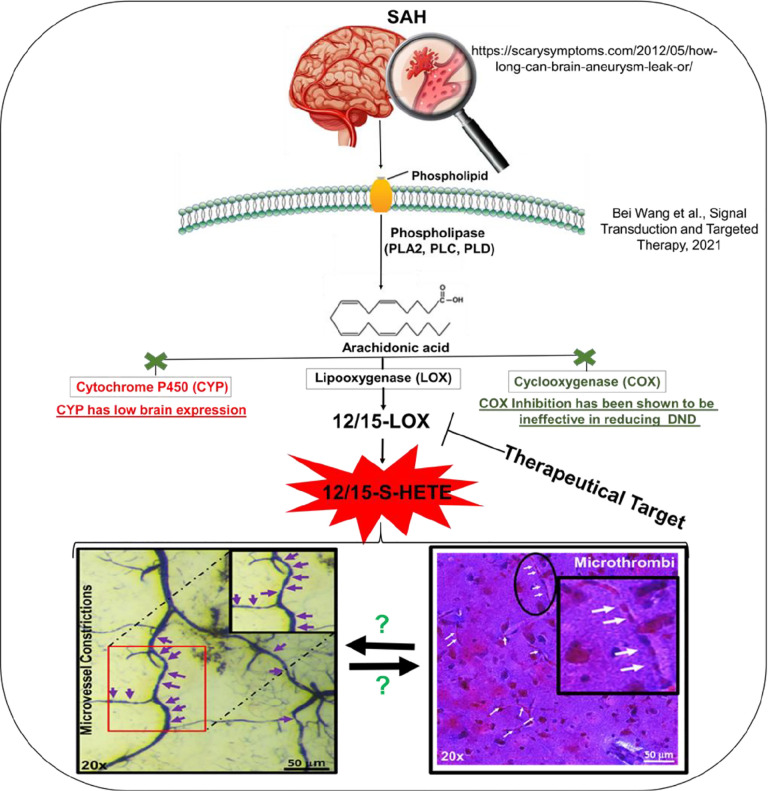
Schematic of the role of 12/15-Lipooxygenase in inducing microthrombi and microvessel constrictions after SAH.

By discovering the critical role of 12/15-LOX, an inflammation regulator,^16^ in microvessel constrictions and platelet activation, our data suggests that effective inhibition of 12/15-LOX may lead to a reduction of platelet activation, microthrombi and small vessel constrictions (Fig. 12), ultimately contributing to the reduction of DCI after SAH.

### Limitations

This study is not without limitations. First, ML351 is cell-permeable and appears to be non-reductive and reversible.^52^ ML351 exhibits greater than 250-fold selectivity towards its primary target, 12/15-LOX (IC50 = 200nM), compared to related LOX isoenzymes such as 5-LOX. However, we cannot exclude the potential protective effect of inhibited 5-LOX with ML351 (IC50 = 50μM) on the outcome.^52,53^ ML351 inhibits 12/15-LOX throughout the body. Our data suggests that 12/15-LOX expression is increased in endothelial and pericyte cells, and Gaberel *et al*., reports an increased expression of 12/15-LOX in macrophages.^15^ Thus we cannot state whether the observed effects are due to one cell type or a combination; future studies should investigate selectively targeting 12/15-LOX within these cell types to identify which cell(s) are key regulators of SAH pathophysiological events.

Second, a component of the vehicle used for ML351, Cremophor EL, possesses a documented neuroprotective effect.^54^ In this study, we also observed that the vehicle may reduce platelet activity after SAH (Supplement Fig. 4). Yet, ML351 clearly has a protective effect since it significantly improved several outcomes compared to vehicle-treated mice. The protective effect by Cremophor EL, especially on platelets (Supplemental Fig. 4) may be an explanation for why female mice with SAH did not have elevated microthrombi counts (compared to sham) in this study which is different from our other studies.^55^

As mentioned above, there are multiple mechanisms of metabolizing arachidonic acid. In this study we only examined 12/15-LOX. When 12/15-LOX is inhibited, arachidonic acid may be metabolized through other ways as 5-LOX, metabolizing arachidonic acid to 5-HPETE which can be further metabolized to n-HETE, balancing out the missing HETE from 12/15-LOX.^43^ Also mentioned above, the arachidonic acid may also be metabolized by COX which also contributes vasoconstrictors and pro-inflammatory metabolites.^43,56^ Additional studies are needed to examine the effect of 12/15-LOX inhibition on arachidonic acid metabolism and arachidonic acid enzyme function.

Another limitation of this study is that we only used a single dose administered at an early time-point after SAH. Since there were mixed beneficial effects, especially in females, we may not have used the optimal dosing regimen. Testing other doses or timing of administration would aid in not only identifying the best regimen, but would also help to determine if there are sex-specific benefits by ML351. Furthermore, no long-term study was conducted.

## Conclusion

In this study, we observed that inhibiting 12/15-LOX leads to a reduction in delayed microvessel constriction and microthrombi formation, enhances cerebral perfusion, and consequently results in less neurological deficits and DCI. While interference with the arachidonic acid-COX pathway has seen limited clinical translation, this study, along with the work by Gaberel *et al*,^15^ indicates that arachidonic acid metabolism by 12/15-LOX may represent a crucial mechanism following SAH.

## Data Availability

Data is provided within the manuscript and supplementary information file. All raw data and materials are available from the corresponding author on request.
